# Treatment patterns of antidiabetic and kidney protective therapies among patients with type 2 diabetes mellitus and chronic kidney disease in Colombia. The KDICO descriptive study

**DOI:** 10.1186/s13098-023-01126-6

**Published:** 2023-07-04

**Authors:** Manuel E Machado-Duque, Andres Gaviria-Mendoza, Luis F Valladales-Restrepo, Juan Sebastian Franco, Maria de Rosario Forero, David Vizcaya, Jorge E Machado-Alba

**Affiliations:** 1 Grupo de Investigación en Farmacoepidemiología y Farmacovigilancia, Universidad Tecnológica de Pereira-Audifarma S.A, Pereira, Risaralda 660003 Colombia; 2 Grupo de Investigación Biomedicina, Facultad de Medicina, Fundación Universitaria Autónoma de las Américas, Pereira, Colombia; 3 Bayer AG, Pereira, Colombia; 4 Bayer AG, Barcelona, Spain

**Keywords:** Diabetes Mellitus, type 2, Chronic kidney failure, Metformin, Dipeptidyl-peptidase IV inhibitors, Angiotensin receptor antagonists, Sodium-glucose transporter 2 inhibitors, Glucagon-like peptide 1, Pharmacoepidemiology

## Abstract

**Background:**

Type 2 diabetes mellitus is one of the most common causes of chronic kidney disease (CKD) worldwide and prevalence of 1.75 per 100 inhabitants in Colombia. The aim of this study was to describe the treatment patterns of a group of patients with type 2 diabetes mellitus and CKD in an outpatient setting from Colombia.

**Methods:**

A cross-sectional study in adult patients with type 2 diabetes mellitus and CKD identified in the Audifarma S.A. administrative healthcare database between April 2019 and March 2020 was performed. Sociodemographic, clinical and pharmacological variables were considered and analyzed.

**Results:**

A total of 14,722 patients with type 2 diabetes mellitus and CKD were identified, predominantly male (51%), with a mean age of 74.7 years. The most common treatment patterns of type 2 diabetes mellitus included the use of metformin monotherapy (20.5%), followed by the combination of metformin + dipeptidyl peptidase-4 inhibitor (13.4%). Regarding the use of drugs with nephroprotective properties, the most prescribed treatments were angiotensin receptor blockers (67.2%), angiotensin converting enzyme inhibitors (15.8%), sodium glucose cotransporter 2 inhibitors (SGLT2i) (17.0%) and glucagon-like peptide-1 analogs (GLP1a) (5.2%).

**Conclusion:**

In Colombia, the majority of patients with type 2 diabetes mellitus and CKD identified in this study were treated with antidiabetic and protective medications to ensure adequate metabolic, cardiovascular, and renal control. The management of type 2 diabetes mellitus and CKD may be improved if the beneficial properties of new groups of antidiabetics (SGLT2i, GLP1a), as well as novel mineralocorticoid receptor antagonists, are considered.

**Supplementary Information:**

The online version contains supplementary material available at 10.1186/s13098-023-01126-6.

## Introduction

Type 2 diabetes mellitus is a major challenge for the health care system globally and locally and is expected to reach 693 million patients diagnosed worldwide with this disease by 2045 [1, 2]. Patients with type 2 diabetes mellitus are at higher risk for microvascular and macrovascular complications, resulting in a heavy economic burden for society [1]. An estimated 20–40% of type 2 diabetes mellitus patients develop chronic kidney disease (CKD), which is characterized by progressive damage and irreversible loss of function in the kidney, eventually resulting in kidney failure [3]. In fact, type 2 diabetes mellitus and hypertension are the leading causes of CKD in high-, middle- and low-income countries [4].

In Colombia, CKD has been identified as a high-cost disease due to its increasing prevalence and incidence, as well as its high risk of complications and mortality. As a consequence, this disease leads to a high consumption of resources from the health care system through loss of work capacity and decrease in quality of life. According to the 2021 High Cost Account report, it is estimated that a total of 1.576.508 million people have type 2 diabetes mellitus, of whom 40% have been diagnosed with CKD in Colombia [5]. Although the incidence and prevalence of CKD in Colombia have remained stable (3.05 per 1,000 inhabitants and 1.75 per 100 inhabitants, respectively), with the majority of patients diagnosed in stage 1–2 (45%) and stage 3 (34%), there has been an increase in mortality (74.44 per 100,000 inhabitants) in Colombia [5].

Therefore, it is imperative to manage and intervene in CKD and its avoidable outcomes in patients with type 2 diabetes mellitus [6]. Alongside dietary and lifestyle interventions, current proven pharmacological strategies for CKD prevention and treatment in type 2 diabetes mellitus patients include optimization of glycemic control, blood pressure and blood lipid levels [7, 8]. Treatment with renin–angiotensin system inhibitors, such as angiotensin converting enzyme inhibitors (ACEis) and angiotensin receptor blockers (ARBs), is regarded as the standard of care (SoC) treatment for patients with CKD [9]. ACEis have demonstrated a significant reduction in the risk of cardiovascular morbidity and mortality in patients with early CKD (stage 1 and 2) but have not been evaluated in patients with stage 3 and 4 CKD with concomitant type 2 diabetes mellitus [10]. In addition, studies with ARBs have not demonstrated their impact on the improve on overall survival of patients with CKD and type 2 diabetes mellitus and have shown a positive impact on patients with high urine albumin-to-creatinine ratio (UACR) (≥ 300 mg/g) [9, 11]. More recently, sodium glucose cotransporter 2 inhibitors (SGLT2i) were able to demonstrate reductions in cardiac and renal outcomes in patients with CKD and type 2 diabetes mellitus and UACR > 300 mg/g or > 200 mg/g [12, 13].

However, despite these recent advances, more than 10% of patients may experience progression of CKD and cardiovascular events, highlighting the persistence of the risk of deterioration in this population [14]. The pathophysiology underlying CKD in type 2 diabetes mellitus is complex, and there are multiple factors involved in the progression of CKD and its associated morbidity [5]. The Colombian Healthcare System offers universal coverage through two affiliation regimes, one with payment by employers and workers, and another subsidized by the state, both offer a benefit plan that includes antidiabetics, such as metformin, SGLT2i, glucagon-like peptide-1 analogs (GLP1a), and insulins, among others. Understanding the patient profiles of the different treatment choices is fundamental to offer a comprehensive clinical approach to type 2 diabetes mellitus and CKD. Thus, the objective of this study was to describe the pharmacologic treatment patterns among a group of ambulatory patients with type 2 diabetes mellitus and CKD in a real-world setting in Colombia.

## Methods

A descriptive, retrospective cross-sectional study with the aim of characterizing the treatment patterns of patients with type 2 diabetes mellitus and CKD was conducted using a population database of medication dispensing from Audifarma S.A., the largest logistics operator in the country, with data from more than 8.5 million people affiliated with the Colombian Healthcare System in six different health insurance companies. All patients 18 years and older with at least one year of enrollment with their insurance provider (contributory regime (contribution by the worker and employer to the health system)) and at least one year of available data, with a diagnosis of both type 2 diabetes mellitus and CKD using International Classification of Diseases 10 (ICD-10) codes (E110-E119, E140-E149, N180-N185, N188-N190, Y841, Z490-Z492) and medication dispensing identified in the database between April 1st, 2019, and March 31st, 2020, were included. Each medication that is registered in the database was effectively dispensed to the patient to whom it was delivered.

From the medication consumption information for the population meeting the inclusion criteria, a database was constructed containing the following data:

- Sociodemographic: Sex, age (recorded at the time of first dispensation), and city and department of care grouped by geographical area according to the regions of Colombia considering the classification of the National Administrative Department of Statistics (DANE) of Colombia, as follows: Bogotá-Cundinamarca region, Caribbean region, Central region, Eastern region, Pacific region and Amazon-Orinoquía region.
- Comorbidities: Diagnoses identified as comorbidities according to ICD-10 codes during the observation period.
- Prescribing physician: The prescribing physician’s specialty.
- Medications used: For each medication, the dosage form and dispensing date were identified. Specific focus on (a) medications used for the management and control of type 2 diabetes mellitus (oral normoglycemic agents, insulins); (b) medications used for the management of type 2 diabetes mellitus and a positive impact on renal function (sodium-glucose cotransporter 2 inhibitors (SGLT2i), glucagon-like peptide-1 analogs (GLP1a)); (c) renin angiotensin aldosterone system (RAAS) blockers (ACEi, ARB, renin inhibitors, ARB + neprilysin inhibitors); (d) mineralocorticoid receptor antagonists (spironolactone, eplerenone).
- Treatment regimens: For each treatment, the following information was obtained: frequency and proportion of use within each therapeutic class (e.g., within ARBs, losartan, valsartan, etc.), mean dose and dosing interval, as well as the defined daily dose (DDD). In addition, the most frequent drug combinations were identified for the treatment of type 2 diabetes mellitus and CKD patients. The frequency and proportion of each combination were obtained.
- Concomitant medications: Medications prescribed for other indications identified during the study period were described.

This database does not include clinical information, and thus, the disease severity, diagnostic test results and other clinical variables were not included.

### Statistical analysis

The data were analyzed with the statistical package SPSS Statistics, version 26.0 for Windows (IBM, USA). A descriptive analysis was performed with frequencies and proportions for qualitative variables and central tendency and dispersion measures for quantitative variables. For these quantitative variables, normality was initially tested using the Kolmogorov–Smirnov test. For variables with normal behavior, the means and standard deviations are presented, and for variables without normality, the medians and interquartile ranges are presented. A multinomial multivariate analysis was performed, seeking to identify possible variables associated with receiving at least one RAAS blocker or SGLT2i versus receiving neither in this population, and receiving the combination of both, which is defined as complete nephroprotective treatment, versus receiving no treatment. Sociodemographic variables, comorbidities, and concomitant medications were included in the regression. A significant p value less than 0.05 was considered.

### Ethical considerations

The protocol received endorsement from the Bioethics Committee of the Universidad Tecnológica de Pereira under the classification of research “without risk” (approval code: 83-081121). According to Resolution 8430 of 1993 of the Ministry of Health of Colombia, risk-free research does not require the signing of an informed consent if it is information obtained from databases or clinical records. The research was performed with the authorization of Audifarma S.A., the drug dispensing company that owns the database. The principles for the confidentiality of information established by the Declaration of Helsinki were observed.

## Results

Among the 194,023 patients with a primary diagnosis of type 2 diabetes mellitus, a total of 14,722 (7.5%) were identified to have concomitant CKD during the observation period in the database. Of these 14,722 patients with type 2 diabetes mellitus and CKD, 51% were men, and the mean age was 74.7 years. Regarding the distribution of patients by region in Colombia, those with the greatest number of patients were from the Pacific, Central, and Bogotá-Cundinamarca regions. Arterial hypertension, hypothyroidism and dyslipidemia were identified as the main comorbidities. Table 1 shows the main sociodemographic characteristics and comorbidities in the evaluated population. Statins, followed by acetylsalicylic acid, acetaminophen, and proton pump inhibitors, were the main comedications used by these patients (see Supplementary Table 1).

**Table 1:** Sociodemographic characteristics and comorbidities of a group of patients with type 2 diabetes mellitus and chronic kidney disease in Colombia

Variable	Frequency	%
Age (mean; SD)	74.7 ± 10.9	
Male gender	7513	51.0
Age group		
<50 years	313	2.1
50 to 59	965	6.6
60 to 69	3040	20.6
70 to 79	5155	35.0
80 to 89	4235	28.8
> 90	1014	6.9
Region		
Pacific	7169	48.7
Central	2719	18.5
Bogota/Cundinamarca	2327	15.8
Caribbean	2049	13.9
Oriental	451	3.1
Amazon	7	0.0
Comorbidities		
Arterial hypertension	10,665	72.4
Hypothyroidism	1138	7.7
Dyslipidemia	989	6.7
Chronic obstructive pulmonary disease	596	4.0
Depression	569	3.9
Dementia	431	2.9
Chronic pain	388	2.6
Anxiety	275	1.9
Obesity	261	1.8
Acute myocardial infarction	231	1.6
Osteoporosis	218	1.5
Atrial fibrillation	136	0.9
Peripheral vascular disease	133	0.9
Parkinsonism	81	0.6
Psychotic disorders	48	0.3
Valve disease	21	0.1
Congestive heart failure	19	0.1

SD: standard deviation

### Treatment patterns of type 2 diabetes mellitus and CKD

Regarding the treatment for type 2 diabetes mellitus in the cohort of patients with CKD, it was possible to identify that the most commonly used drugs were metformin alone or associated with some other antidiabetic, followed by dipeptidyl peptidase-4 (DPP4) inhibitors (especially linagliptin and sitagliptin), some type of insulins (particularly long-acting and short-acting analogs), and sulfonylureas. In the group of drugs with a positive impact on kidney function, the use of SGLT2i (empagliflozin being the most frequent) and GLP1a (especially liraglutide) was notable. Table 2 shows the prescription patterns of the different antidiabetic drugs, with their frequencies, doses, relationship between the mean dose and the DDD, and the proportion by sex for each one.

**Table 2:** Patterns of antidiabetic drug use for a group of patients with type 2 diabetes mellitus and chronic kidney disease in Colombia

Name	Frequency	%	Average Dose ± DE	nDDD	Ratio M:F	Age (average)
Biguanides						
Metformin	9901	67.3	1376.2 ± 631.7	1.5	49.6	74.5 ± 10.6
DPP4 inhibitors	7691	52.2			52.9	74.7 ± 10.7
Linagliptin	3244	22	5.09 ± 0.67	1.01	57.5	75.2 ± 10.79
Linagliptin/Metformin	673	4.6	5.09 / 1703.7 a	1.01		
Sitagliptin	1301	8.8	90.3 ± 23.7	1.1	50.1	73.3 ± 10.1
Sitagliptin/Metformin	1409	10.1	90.3 / 1798.5 a	1.1		
Vildagliptin	666	4.5	83.7 ± 23.5	0.8	50.2	73.6 ± 10.6
Vildagliptin/Metformin	737	5	83.7 / 1755.3 a	0.8		
Saxagliptin	70	0.5	5.04 ± 1.34	1	39.8	73.2 ± 10.2
Saxagliptin/Metformin	264	1.8	5.04 / 1814.4 a	1		
SGLT2 inhibitors	2500	17.0			54.8	73.5 ± 11.4
Empagliflozin	1488	10.1	20.01 ± 7.3	1.14	57.4	71.2 ± 10.5
Empagliflozin/Metformin	360	2.4	22.4 / 1755	1.28		
Empagliflozin/Linagliptin	43	0.3	18.4 / 5.5	1.05		
Dapagliflozin	563	3.6	10.6 ± 1.7	1.06	48.9	68.5 ± 10.4
Dapagliflozin/Metformin	177	1.2	10.2 / 1593.2	1.02		
Canagliflozin	1	0.006	300 ± NA	1.5	0.0	68.0 ± NA
GLP-1 analogs	766	5.2			48.3	69.3 ± 10.5
Liraglutide	588	4	1.74 ± 0.42	1.16	47.6	69.0 ± 10.5
Dulaglutide (weekly)	122	0.8	2.54 ± 0.66 b	2.26	57.4	70.8 ± 9.8
Exenatide (weekly)	73	0.5	2 ± NA	0.99	55.7	69.6 ± 10.3
Semaglutide (weekly)	3	0.02	0.58 ± 0.38 b	0.77	66.7	64.0 ± 9.8
Lixisenatide, mcg	3	0.02	20 ± NA	1	66.7	74.6 ± 5.0
Sulfonylureas						
Glibenclamide	210	1.4	7.3 ± 3.4	0.73	42.9	74.3 ± 10.0
Glimepiride	165	1.1	3.2 ± 1.4	1.6	40.6	70.9 ± 10.4
Gliclazide	89	0.6	66.0 ± 18.2	1.1	65.2	69.1 ± 10.5
Insulins (pooled)						
Long action analogs	4757	32.3	NA		54.7	72.1 ± 11.3
Fast action analogs	2477	16.6	NA		54.7	71.1 ± 11.9
NPH	243	1.7	NA		55.1	72.2 ± 11.8
Crystal clear	0	0	NA		NA	NA

SD: standard deviation; nDDD: ratio between the mean dose and the defined daily dose; M:F: masculine: feminine; DPP4: dipeptidyl peptidase 4; SGLT2: sodium-glucose cotransporter type 2; GLP-1: glucagon-like peptide type 1

Regarding the use of drugs with nephroprotective properties, the most prescribed treatments were ARB (especially losartan), followed by ACEi (particularly enalapril) in 67.2% and 15.8%, respectively; in addition, a significant proportion of patients were receiving other antihypertensive drugs such as calcium channel blockers (amlodipine), β-blockers (especially metoprolol and carvedilol) and mineralocorticoid receptor antagonists (spironolactone) (see Table 3).

**Table 3:** Patterns of antihypertensive and nephroprotective agents use for a group of patients with type 2 diabetes mellitus and chronic kidney disease in Colombia

Medication Name	n	%	Average Dose ± DE	nDDD	Male Ratio (%)	Age (average)
Angiotensin II receptor blocker	9895	67.2			49.2	75.4 ± 10.5
Losartan	7857	53.4	97.5 ± 37.6	1.9	48.6	75.5 ± 10.5
Losartan/Hydrochlorothiazide	971	6.6	99.5 / 22.7	2	48.6	75.5 ± 10.5
Irbesartan	603	4.1	261.5 ± 73.6	1.7	60.2	72.3 ± 11.3
Valsartan	561	3.8	225.8 ± 89.4	2.8	45.9	74.2 ± 9.2
Valsartan/Hydrochlorothiazide/Amlodipine	219	1.5	216.3 / 16.1 / 8.9	2.7	45.9	74.2 ± 9.2
Irbesartan/Amlodipine	193	1.3	280.5 / 8.6	1.9		
Telmisartan	158	1.1	87.3 ± 29.1	2.2	49	76.0 ± 10.2
Telmisartan/Amlodipine	85	0.6	90.3 / 9.1	2.3		
Candesartan	74	0.5	21.9 ± 11.1	2.7	56.9	73.5 ± 10.9
Irbesartan/Hydrochlorothiazide	53	0.4	280.5 / 18.8	1.9		
Valsartan/Hydrochlorothiazide	48	0.3	216.3 / 16.1	2.7		
Losartan/Amlodipine	28	0.2	99.5 / 7.3	2		
Olmesartan	23	0.2	38.2 ± 9.4	1.9	55.6	74.5 ± 11.3
Telmisartan/Hydrochlorothiazide	16	0.1	90.3 / 17.2	2.3		
Olmesartan/Amlodipine	15	0.1	38.2 / 8.7	1.9		
Eprosartan	8	0.1	600 ± NA	1	50	79.5 ± 12.2
Olmesartan/Hydrochlorothiazide	7	0.04	38.2 / 23.2	1.9		
Candesartan/Hydrochlorothiazide	5	0.03	32 / 17.5	4		
Angiotensin converting enzyme	2325	15.8			56.6	73.5 ± 11.4
Enalapril	2213	15	21.6 ± 15.2	2.1	57.3	73.3 ± 11.4
Captopril	110	0.7	71.6 ± 31.9	1.4	40.4	77.6 ± 10.7
Lisinopril	12	0.07	21.7 ± 9.4	2.1	33.3	77.8 ± 12.0
Perindopril/Indapamide	9	0.04	8.88 / 7.5	2.2	62.5	74.2 ± 9.7
Perindopril/Amlodipine	6	0.06	8.88 / 2.2	2.2	62.5	74.2 ± 9.7
Quinapril	2	0.01	30 ± NA	2	100	78.5 ± 2.2
Perindopril	1	0.01	8.88 ± NA	2.2	62.5	74.2 ± 9.7
Mineralocorticoid receptor antagonist	1598	10.9			58.4	73.9 ± 11.2
Spironolactone	1575	10.7	27.5 ± 13.6	0.36	58.3	73.9 ± 11.2
Eplerenone	28	0.2	35.4 ± 14.3	0.7	71.4	74.1 ± 10.1
Calcium channel blockers						
Amlodipine	4043	54.9	6.3 ± 2.2	1.26	50.9	74.8 ± 10.5
Nifedipine (retard)	966	6.6	34.4 ± 10.6	1.14	47.3	75.7 ± 10.4
Verapamil	471	3.2	175.7 ± 56.8	0.24	36.1	75.1 ± 9.1
β-blockers						
Metoprolol	2276	15.5	86.8 ± 35.7	0.58	49.2	75.5 ± 9.9
Carvedilol	2063	14	21.6 ± 13.4	0.57	58.1	74.8 ± 11.8
Nebivolol	28	0.2	4.6 ± 2.6	0.92	53.6	75.3 ± 7.5
Propranolol	93	0.6	56.7 ± 26.5	0.35	50.9	74.1 ± 9.6

SD: standard deviation; nDDD: ratio between the mean dose and the defined daily dose

The most common treatment patterns of type 2 diabetes mellitus included the use of metformin monotherapy, followed by the combination of metformin + DPP4 inhibitor or metformin + DPP4 inhibitor + insulin, and the use of a DPP4 inhibitor + insulin. However, the finding of 8.9% of cases in which antidiabetic therapy could not be identified during the observation period was striking (Table 4). Regarding antihypertensive drugs, 19.3% of the patients were receiving monotherapy with an ARB, ACEi, β-blocker or calcium channel blocker. The most common combinations were ARB + calcium channel blocker or an ARB + calcium channel blocker + hydrochlorothiazide + furosemide. Approximately 10% of the patients with arterial hypertension had not reported any antihypertensive medication use (Table 4). In addition, a total of 80 (0.5%) patients without antidiabetic or antihypertensive prescriptions were identified, despite having both diagnoses.

**Table 4:** Antidiabetic and antihypertensive drugs use and combinations of a group of patients with type 2 diabetes mellitus and chronic kidney disease in Colombia

Antidiabetic drugs alone or in associations	Frequency	%
Metformin	3019	20.5
Metformin + dipeptidyl peptidase-4 inhibitor	1976	13.4
Metformin + dipeptidyl peptidase-4 inhibitor + Insulin	1500	10.2
No treatment for diabetes mellitus	1303	8.9
Dipeptidyl peptidase-4inhibitor + Insulin	1052	7.1
Dipeptidyl peptidase-4 inhibitor	934	6.3
Insulin	741	5
Metformin + dipeptidyl peptidase-4 inhibitor + Sodium-Glucose Transporter 2 Inhibitors + Insulin	639	4.3
Metformin + dipeptidyl peptidase-4 inhibitor + Sodium-Glucose Transporter 2 Inhibitors	427	2.9
Metformin + Sulfonylureas	412	2.8
48 other combinations	2719	18.5
**Antihypertensive drugs alone or in associations**	**Frequency**	**%**
ARBs	1611	10.9
Without antihypertensive therapy	1518	10.3
ARBs + Calcium channel blocker	1170	7.9
ARBs + Calcium channel blocker + Hydrochlorothiazide + Furosemide	902	6.1
ARBs + Calcium channel blocker + β-blocker + Hydrochlorothiazide + Furosemide	792	5.4
ACEis	761	5.2
ARBs + Calcium channel blocker + Hydrochlorothiazide	659	4.5
ARBs + β-blocker	621	4.2
ARBs + Calcium channel blocker + β-blocker	588	4
ARBs + β-blocker + Hydrochlorothiazide + Furosemide	532	3.6
ARBs + Hydrochlorothiazide + Furosemide	528	3.6
ARBs + Hydrochlorothiazide	488	3.3
β-blocker	292	2
ARBs + Calcium channel blockers + β-blocker + Hydrochlorothiazide	277	1.9
ARBs + Calcium channel blockers + β-blocker + Hydrochlorothiazide + Furosemide +	226	1.5
Mineralocorticoid receptor antagonist		
ARBs + β-blocker + Hydrochlorothiazide + Furosemide + Mineralocorticoid receptor antagonist	185	1.3
ARBs + β-blocker + Hydrochlorothiazide	181	1.2
Calcium channel blocker	174	1.2
ACEis + β-blocker	154	1
ACEis + Hydrochlorothiazide	150	1
Other 76 combinations	2913	19.8

ARBs: angiotensin receptor blockers. ACEis: angiotensin converting enzyme inhibitors

Table 5 shows the results of the multinomial logistic regression, after adjustment, variables associated with a higher and lower probability of using a SLGT2i or RAAS blocker versus using none and similarly versus receiving complete nephroprotective treatment.

**Table 5:** Multinomial logistic regression to identify variables that were associated with a greater probability of receiving RAAS blocker or SGLT2 inhibitor versus receiving neither in this population, and receiving the combination of both (complete nephroprotective treatment)

	Variable	B	Sig^a^	OR ^b^	95% CI ^c^	
					Lower	Upper
SLGT2^d^ Inhibitor or RAAS^e^ blocker	Intersection	-0.57				
	Gender: male	-0.11	0.02	0.90	0.82	0.98
	Age (years)	0.01	0.00	1.01	1.01	1.02
	Hypertension	0.85	0.00	2.34	2.13	2.58
	Obesity	0.54	0.02	1.72	1.11	2.66
	Use of aspirin	0.66	0.00	1.93	1.76	2.12
	Use of β-blockers	0.16	0.00	1.18	1.06	1.31
	Use of Proton Pump Inhibitors	0.10	0.04	1.11	1.01	1.22
	Use of metformin	0.33	0.00	1.39	1.27	1.53
	Use of insulin	-0.17	0.00	0.85	0.77	0.93
	Be treated in Bogota-Cundinamarca region	ref	ref	ref	Ref	ref
	Be treated in the Caribe region	0.05	0.51	1.06	0.90	1.24
	Be treated in the Central region	-0.16	0.04	0.85	0.73	0.99
	Be treated in the Oriental region	-0.04	0.80	0.96	0.74	1.27
	Be treated in the Pacific region	0.25	0.00	1.28	1.12	1.47
Complete treatment (ACEi^f^ or ARB^g^ + SGLT2i)	Intersection	-1.29				
	Gender: male	0.03	0.67	1.03	0.91	1.16
	Age (years)	-0.02	0.00	0.98	0.98	0.99
	Dementia	-0.54	0.01	0.58	0.38	0.88
	Dyslipidemia	0.65	0.00	1.91	1.52	2.40
	Hypertension	0.97	0.00	2.63	2.29	3.03
	Obesity	1.15	0.00	3.14	1.96	5.05
	Use of aspirin	0.93	0.00	2.53	2.23	2.88
	Use of β-blockers	0.25	0.00	1.28	1.12	1.47
	Use of Proton Pump Inhibitors	0.15	0.02	1.17	1.03	1.32
	Use of metformin	0.61	0.00	1.83	1.61	2.08
	Use of insulin	0.97	0.00	2.63	2.31	3.00
	Be treated in Bogota-Cundinamarca region	ref	ref	ref	Ref	ref
	Be treated in the Caribe region	-0.28	0.02	0.76	0.60	0.96
	Be treated in the Central region	0.13	0.22	1.14	0.93	1.39
	Be treated in the Oriental region	0.13	0.50	1.13	0.79	1.63
	Be treated in the Pacific region	0.54	0.00	1.72	1.44	2.07

^a^ significance level. ^b^ OR: Odds ratio. ^c^ 95% confidence interval. ^d^ SGLT2: sodium-glucose cotransporter type 2; ^e^ RAAS blocker: renin angiotensin aldosterone system blocker. ^f^ ACEis: angiotensin converting enzyme inhibitors. ^g^ ARBs: angiotensin receptor blockers

## Discussion

Treatment patterns in patients with type 2 diabetes mellitus and concomitant diagnosis of CKD were identified, allowing us to describe the use of antidiabetics and antihypertensives with nephroprotective properties. This information has not been described for such a large population in Colombia, which provides real world data, useful for physicians, patients, and decision-makers, in such a way to allow optimization of therapy and identification of patients with potential prescription omissions.

The prevalence of CKD found in the group of patients with type 2 diabetes mellitus (7.1%) slightly exceeds the one reported by a study that collected information from 37 countries (6.1%), which had a mean evolution of the condition of 5.7 years and an age of only 56 years [15]; however, it is lower than that reported by the study by Lisa Chu et al. in Canada (47.9%), with the difference that the latter also included cases with stages 1 and 2 with micro- or macroalbuminuria, which could not be identified in this analysis, and maybe a reflection of the recording of data from patients with more advanced stages by Colombian doctors or a lack or underreporting of the diagnosis [16]. According to a global registry published in 2022 that estimated the worldwide prevalence at 9.1%, this information could represent an underreported diagnosis of CKD in Colombia, added to the fact that only patients with a baseline diagnosis of type 2 diabetes mellitus were included in this analysis, a situation explained by the origin of the data from a medication dispensing database, in which only ICD-10 diagnoses related to prescription [17] were considered. Additionally, in Colombia, the High-Cost Account that quantifies the diseases that generate the greatest economic burden for the country estimates that the prevalence is even higher than that of the global report [5].

The mean age identified in this cohort of patients was over 74 years, which is similar to that identified in patients from the United States, implying a long evolution of the disease that has led to microvascular complications [18], as when compared with a cohort of Colombian patients with type 2 diabetes mellitus without defined complications, the mean age was approximately 65 years [15, 19].

A finding of interest from the pharmacoepidemiological point of view were the differences in the probability of receiving complete nephroprotective treatment, this being more likely in the Pacific region compared to Bogotá, and the Caribbean region less likely to receive nephroprotection, which is somewhat explained by differences in access to health systems between regions, different medical practices and medical schools in each region, a situation previously described in Colombia and the world [20, 21].Arterial hypertension was the most common concomitant clinical condition in this subgroup of patients with type 2 diabetes mellitus and CKD, consistent with the findings of the High-Cost Account of Colombia [5], Comorbidity that also increased the probability in these patients with diabetes mellitus and kidney damage of using some of the nephroprotective therapies, this being expected as the RAAS blocker is antihypertensive drugs and thus being able to provide better protection against those who are not hypertensive, also making evident the lack of knowledge regarding the use of medications as nephroprotectors [22], and the lack of their use in this particular indication [23]. The low proportion of records of ischemic heart disease and heart failure is striking, possibly related to the underreporting of physicians when prescribing medications. Notably, advanced age, type 2 diabetes mellitus and high blood pressure are the most important risk factors for the development of CKD and for the need for hemodialysis [24, 25]. The abovementioned highlights the importance of conducting adequate metabolic control with lifestyle changes and the use of antidiabetic medications that also have a positive impact on the progression of kidney damage, added to cardiovascular treatment that also protects kidney function [26].

Of note, 8.9% of patients with diagnoses of type 2 diabetes mellitus and CKD were not receiving antidiabetic therapy during the observation period, which could be related to their lack of attendance at medical check-ups, to the need to purchase medications with pocket money, to the lack of follow-up or even to not being prescribed an antidiabetic medication. However, the vast majority did receive treatment, especially with metformin, although its identification in only 67.3% of patients is lower than expected according to the current recommendations at the time the analysis was made. Almost all patients should receive treatment according to the clinical practice guidelines [9, 27], with certain exceptions, such as having a glomerular filtration rate of less than 30 mL/min, a possible situation present in this group of patients, but which could not be determined due to the lack of this information in the database from which the information was obtained [28].

This cohort of patients more frequently received some DPP4 inhibitor in addition to metformin, which are recommended in first and second line in different clinical scenarios; however, current evidence suggests that the best combination in patients who also have CKD is metformin plus an SGLT2i [9], a therapy that only 17% of patients received; or metformin plus a GLP1a, which was observed in only 5.2% of cases, as these treatments have shown a benefit on renal function and reduction of proteinuria [12, 29–33]. This low proportion of use may be related to access difficulties, lack of up-to-date knowledge of physicians, and clinical inertia, in addition to the contraindications of antidiabetics or the control of patients with monotherapy. However, these findings should motivate further research to identify the causes and thus propose strategies to ensure that patients with type 2 diabetes mellitus and coexisting CKD receive the most appropriate treatment. In addition, more recent evidence provides data in favor of using finerenone, a nonsteroidal aldosterone antagonist, which has shown benefits in slowing the progression of CKD and favorable cardiovascular impact [7, 8], which is currently unavailable in Colombia but opens new perspectives regarding the optimal therapy for the management of this condition. Finally, comparing the use of antidiabetic therapies with renal benefit [34, 35], the study by Chu et al. in Canada showed that 47.6% of patients with this dual condition received any type of SGLT2i and up to 29.6% received a GLP1a [16], and although in Colombia all antidiabetic drugs are covered by the Health System [36], the high cost, especially of GLP1a, as well as the lack of cost-effectiveness and cost-utility analyses, make it difficult to make decisions to ensure that a greater number of people benefit from the properties of these products.

The cornerstone of CKD management is adequate control of arterial hypertension in addition to metabolic control of type 2 diabetes mellitus with medications that block the RAAS, such as an ACEi or an ARB. These treatments reduce peripheral vascular resistance and inhibit aldosterone, thus avoiding sodium and water retention and therefore volume overload but also generate vasodilation of the efferent arterioles of the kidney, effects known since 1992, when in the Collaborative Study Group (CSG) Captopril Trial, the benefit of protecting the deterioration of renal function was demonstrated [37]. Likewise, the Heart Outcome Prevention Evaluation (HOPE) study with ramipril showed a decrease in morbidity and mortality in those suffering from type 2 diabetes mellitus [38]. A network meta-analysis involving patients treated with ACEis and ARBs concluded that enalapril may be one of the most effective therapies for reducing albuminuria [39]. In 2001, the RENAAL study with losartan demonstrated its ability to reduce the progression of CKD and proteinuria [10], while the IDNT studies with irbesartan and MARVAL with valsartan showed these same findings, supporting the fact that it is an effect of the entire pharmacological group [12, 40].

In this analysis, approximately 90% of the patients received a RAAS blocker, either losartan or enalapril, which must provide the antihypertensive effects added to the protectors on renal function, as has been described [26, 41]. The above findings differ from the work published by Zhang YQ et al. in China, where 76.9% of patients with type 2 diabetes mellitus received antihypertensive treatment, mainly with calcium channel blockers (26.6%) and ARB (26.6%) [42], but they are similar to those reported by Zhang J et al. in Australia, where 95.7% were on antihypertensive treatment, and of these 69% were using some RAAS blockers, including the possibility of receiving a mineralocorticoid receptor antagonist, and in this case, all were blockers of steroid origin [19, 43].

Finally, some comedications such as NSAIDs were used with a high frequency, which increases the individual risk of kidney damage [44], in addition to other medications such as PPIs, which are associated with a greater incident risk of CKD and its progression [45, 46], a situation that prescription of one of these such as NSAIDs, leads to prescribing a PPI, and thus jointly potentiate kidney damage, likewise it was found in this study that the use of PPI increased the probability with an OR of 1.28 of using complete nephroprotective therapy, probably by identifying a higher-risk patient, leads to more frequent prescription of drugs and polypharmacy. Evidencing the need to increase knowledge of drugs in physicians treating patients with CKD, avoiding the use of risk therapies and promoting the use of those with benefit.The main limitations of this analysis are recognized as the observational nature, with information from a drug dispensing database, There is no sociodemographic or clinical information such as ethnicity, economic status, evolution of diabetes mellitus, among others. So it is possible that there is underreporting of the diagnosis of CKD and even diseases of the cardiovascular system as the ICD-10 diagnoses registered in the medication delivery database are associated with some medication prescriptions, In addition, for each drug dispensing, up to two diagnoses are recorded, so there is a potential underreporting of the frequency of comorbidities. In addition, many of the diseases have multiple indications, and there were no data on renal function or evidence of proteinuria, so the state of deterioration could not be classified. There was also no information on the level of metabolic control of type 2 diabetes mellitus or control of arterial hypertension to determine how effective the treatments were. The lockdown and confinement measures in Colombia began on March 25, 2020 [47], therefore, during six days after the end of the observation period of the study, changes could be generated in the patterns of dispensing and prescriptions of medicines. However, the study has some strengths, especially the number of patients included and the rigor that was applied in the search for the dispensing records of each patient, providing this database reliability in other studies and allowing for the development of multiple pharmacoepidemiological studies.

## Conclusion

With the above findings, we concluded that this group of patients with type 2 diabetes mellitus and CKD are adults over 70 years of age, who mostly also suffer from arterial hypertension and are treated mainly with metformin alone or combined with DPP4 inhibitors and insulins for their type 2 diabetes mellitus and with ARB or ACEi for their arterial hypertension and as nephroprotective agents, which can provide adequate metabolic and cardiorenal control. There is a small proportion of cases without pharmacological management, which places them at risk of adverse outcomes and short- and long-term complications. The management of type 2 diabetes mellitus and CKD can be improved if the beneficial properties of new groups of antidiabetics, such as SGLT2i and GLP1a, on cardiovascular outcomes, including renal function, as well as the use of novel mineralocorticoid receptor antagonists, are considered. Additionally, differences were identified in patients receiving nephroprotective therapy between regions of the country. More research is required to delve into the reasons for the selection of certain medications by prescribing physicians, the effectiveness of treatment and especially the impact it may have on macro- and microvascular complications, particularly those related to kidney function.

## Electronic supplementary material

Below is the link to the electronic supplementary material.

Supplementary Material 1

## Data Availability

protocolos.io. **Data access**: DOI: 10.17504/protocols.io.8epv5jkz4l1b/v1 (Private link for reviewers: https://www.protocols.io/private/3E70728FBE8C11ED9CC90A58A9FEAC02 to be removed before publication.)
