# Supplementary material for: Treatment patterns of antidiabetic and kidney protective therapies among patients with type 2 diabetes mellitus and chronic kidney disease in Colombia. The KDICO descriptive study
Source: Diabetol Metab Syndr. 2023 Jul 4;15:150. doi: 10.1186/s13098-023-01126-6 (PMC10318702; doi:10.1186/s13098-023-01126-6)
Supplement: Supplementary file 1 — Supplementary Material 1 [file 13098_2023_1126_MOESM1_ESM.docx]

**Supplementary Table 1.** Most frequently used co-medication of a group of patients with type 2 diabetes mellitus and chronic kidney disease in Colombia.

| **Comedications** | **Frequency** | **%** |
| --- | --- | --- |
| Cardiovascular |  |  |
| Statins | 11551 | 78.5 |
| Acetylsalicylic acid | 8323 | 56.5 |
| Furosemide | 4529 | 30.8 |
| Hydrochlorothiazide | 2706 | 18.4 |
| Fibrates | 1687 | 11.5 |
| Prazosin | 1363 | 9.3 |
| Platelet P2Y12 receptor inhibitors | 749 | 5.1 |
| Direct oral anticoagulants | 436 | 3.0 |
| Warfarin | 148 | 1.0 |
| Analgesics |  |  |
| Acetaminophen | 8248 | 56.0 |
| Nonsteroidal anti-inflammatory | 2592 | 17.6 |
| Opioids | 2534 | 17.2 |
| Endocrine system |  |  |
| Thyroid hormone | 3649 | 24.7 |
| Corticosteroids | 1183 | 8.0 |
| Psychopharmaceuticals |  |  |
| Antidepressants | 2524 | 17.1 |
| Antiepileptics | 1778 | 12.1 |
| Antipsychotics | 693 | 4.7 |
| Benzodiazepines | 196 | 1.3 |
| Others |  |  |
| Proton pump inhibitors | 6349 | 43.1 |
| First generation H1 antihistamines | 1706 | 11.6 |
| Second generation H1 antihistamines | 1582 | 10.7 |
| Ranitidine | 647 | 4.4 |
